# Response of Three Greek Populations of *Aegilops triuncialis* (Crop Wild Relative) to Serpentine Soil

**DOI:** 10.3390/plants10030516

**Published:** 2021-03-10

**Authors:** Maria Karatassiou, Anastasia Giannakoula, Dimitrios Tsitos, Stefanos Stefanou

**Affiliations:** 1 Laboratory of Rangeland Ecology (PO 286), School of Forestry and Natural Environment, Aristotle University of Thessaloniki, 54636 Thessaloniki, Greece; dimitrios.tsitos@gmail.com; 2 Laboratory of Plant Physiology, Department of Agriculture, International Hellenic University, 54700 Sindos, Greece; agianna@ihu.gr; 3 Laboratory of Soil Science, Department of Agriculture, International Hellenic University, 54700 Sindos, Greece; stefst2@ihu.gr

**Keywords:** photosynthesis, water use efficiency, proline, chlorophyll, nickel, nutrient uptake

## Abstract

A common garden experiment was established to investigate the effects of serpentine soil on the photosynthetic and biochemical traits of plants from three Greek populations of *Aegilops triuncialis*. We measured photosynthetic and chlorophyll fluorescence parameters, proline content, and nutrient uptake of the above plants growing in serpentine and non-serpentine soil. The photochemical activity of PSII was inhibited in plants growing in the serpentine soil regardless of the population; however, this inhibition was lower in the Aetolia-Acarnania population. The uptake and the allocation of Ni, as well as that of some other essential nutrient elements (Ca, Mg, Fe, Mn), to upper parts were decreased with the lower decrease recorded in the Aetolia-Acarnania population. Our results showed that excess Ni significantly increased the synthesis of proline, an antioxidant compound that plays an important role in the protection against oxidative stress. We conclude that the reduction in the photosynthetic performance is most probably due to reduced nutrient supply to the upper plant parts. Moreover, nickel accumulation in the roots recorded in plants from all three populations seems to be a mechanism to alleviate the detrimental effects of the serpentine soil stress. In addition, our data suggest that the population from Aetolia-Acarnania could be categorized among the nickel excluders.

## 1. Introduction

Crop wild relatives (CWRs) are an invaluable source of plant genetic materials that can be harnessed through breeding programs to improve stress resistance of cultivated plants [1,2] ensuring food safety [3,4,5]. CWRs have become adapted to various abiotic and biotic stresses to thrive and persist, but today are under risk of extinction in their natural habitat (Directive 92/43 EEC) due to urbanization, scarcity of water, deforestation, desertification, intensive farming, overgrazing, erosion of soil and plant genetic resources, pollution (land, water), and global climate change [2,3,5,6,7]. These species deserve special attention and their habitats need to be protected [8,9]. Seventy five percent of plant biodiversity was lost in the last century, with climate change accounting for a high proportion of loss (16–22%) [10,11]. Climate change can either directly or indirectly influence CWR loss by affecting and endangering crop productivity and promoting the dispersion, establishment, and growth of invasive weeds that outcompete and drive to extinction the native flora [12]. The conservation of CWR biodiversity is currently a major issue that is addressed by different programs and organizations operating internationally [13,14,15].

Stemming from evolutionary forces and leading to survival in a wide range of stressful conditions and environments [8], the genetic diversity of CWRs and their conservation (e.g., collection, protection) is of great importance in our effort to reduce the effects of biotic and abiotic stress factors related to climate change [5]. Interestingly, CWR diversity is highly impacted by climate change but at the same time may provide tools (i.e., genes) for climate change mitigation. The most pronounced results in transferring the resistance to environmental stress to crops come from hybridization of wheat with its wild relatives such as *Aegilops* L., *Agropyron* J. Gaert. and *Haynaldia* Kanitz [5]. Greece is one of the 10 areas with the highest CWR concentration per unit area [2,16] and together with Italy [17,18] is among the most frequently proposed regions for further conservation sampling [1,13].

As stated, species of the genus *Aegilops* have widely been used as gene sources for the improvement of cultivated wheats especially as far as resistance to abiotic and biotic factors is concerned [1,19]. Twelve of these species (e.g., *Aegilops biuncialis* Vis., *Aegilops geniculata* Roth, *Aegilops neglecta* Req. ex Bertol., *Aegilops triuncialis* L. and *Aegilops ventricosa* Tausch) are included in the Greek National Inventory of CWRs. Among them, *Ae. biuncialis* [20], *Ae. ventricose* [18], and *Ae. triuncialis* L., which is native to Mediterranean region and Central Asia [21], are of special interest. Over the last few decades, *Ae. triuncialis* has invaded the low fertility, serpentine soils of California, developing high densities in this edaphically stressful environment that is characterized by high levels of soil toxicity and significant moisture stress [22]. It should be noted that, because of these extreme edaphic conditions, serpentine soil habitats are less prone to invasion than non-serpentine soils [23,24]. Several researchers demonstrated that plant species that can successfully invade serpentine areas may be preadapted to one or more characteristics of these soils [22,25]. In contrast to other Mediterranean countries, such as Italy where several goatgrass species such as *Ae. triuncialis*, *Ae. biuncialis*, and *Ae. ventricosa* grow in annual meadows [26], data regarding growth and existence of Greek *Aegilops* in natural habitats are limited. Greek populations of Aegilops thrive in a variety of environments; however, little information exists on plastic and adaptive responses to abiotic or biotic stress and especially to heavy metals existing in serpentine soils.

Serpentine soils derived from ultramafic rocks are characterized by very low levels of essential macronutrients, high concentration of magnesium (Mg), deficiency of calcium (Ca), low Ca/Mg ratios, high concentrations of heavy metals especially nickel (Ni) and chromium (Cr), and often low water-holding capacity [13,22,25,27]. O’Dell and coworkers [28] demonstrated that among all the unfavorable soil factors of serpentine soils, the imbalanced ratio of Ca/Mg limits the most biomass production. These extreme edaphic conditions define the uniqueness of serpentine soils and the plant species they support [29]. Species growing on these soils can be classified as (a) serpentine-tolerant and (b) serpentine-endemic plants [30]. Heavy metals can be transferred to and concentrated in plant tissues from the soil, and the intake of metals through the food chain by human populations has been widely reported and has raised concerns [31]. These metals have negative effects on the plants themselves and may become a health hazard to living organisms (man and animals) but may also be of concern for the global environment [32].

Plants found in stressful habitats tend to share a suite of traits, or a stress resistance syndrome (SRS), that provides broad adaptation across a range of low productivity, harsh, or edaphically toxic habitats [33,34]. Serpentine soils are an excellent example of a habitat type that selects for genotypes adapted to edaphic stress or “stress tolerance syndrome” [13,35]. Hence, plant populations adapted to serpentine soils are ideal for addressing mechanistic questions of adaptive evolution in nature [36,37]. Plant survival in serpentine soils is a response to several components: chemical, biological, physical, biotic, and temporal [35,38,39]. Additional abiotic stresses may also often exist such as drought, low nutrient cycling rates, and shallow soil depth [40]. For example, there are many studies exploring the importance of drought tolerance to survival in serpentine soils [39,41]. High nickel concentration in plants, often reported in serpentine soils, has an impact on plant growth and produces symptoms of toxicity and often results in competition with other essential metal ions. Excess Ni^2+^ also affects nutrient absorption by roots [42] and inhibits photosynthesis, transpiration, and stomatal conduction [43,44]. Despite the long list of studies addressing aspects of plant response to these stresses, and tolerance to serpentine soils, there is a lack of research for Greek CWR species, and there are no such studies regarding the species *Ae. triuncialis* [30,45,46].

The aim of the current study was to explore both adaptive and plastic aspects of the response of *Ae. triuncialis* (barbed goatgrass) to serpentine soils. Three wild populations of *Ae. triuncialis* all obtained from non-serpentine soils of Western Greece were considered. A suite of traits of the above populations in serpentine and non-serpentine soils under a common garden experimental approach was recorded. We tested the hypotheses that (a) all three populations respond similarly to serpentine and non-serpentine soils regarding physiological and nutritional traits, and (b) serpentine soils negatively affect their performance.

## 2. Results

### 2.1. Description of the Chemical Properties of the Two Soil Types

Organic matter content; pH; and concentrations of macronutrients, micronutrients, and some trace elements in the two types of soils are presented in Table 1. The pH of the serpentine (S) soil was slightly acidic (6.59), while that of the non-serpentine (NS) soil was slightly alkaline (7.57). The concentrations of organic C, total N, and extractable P were higher in the S compared to the NS soil. The exchangeable Ca in the S soil was approximately half of that in NS soil, while the exchangeable Mg was about 2-fold higher in S soil compared to NS (Table 1). The Ca/Mg ratio was approximately 4 times higher in the NS soil. The concentrations of available iron (Fe), manganese (Mn), and Ni were higher in the S soil, while that of exchangeable potassium (K) and sodium (Na) and available copper (Cu) and zinc (Zn) were higher in the NS soil (Table 1).

### 2.2. Gas Exchange Parameters

**Figure 1:**
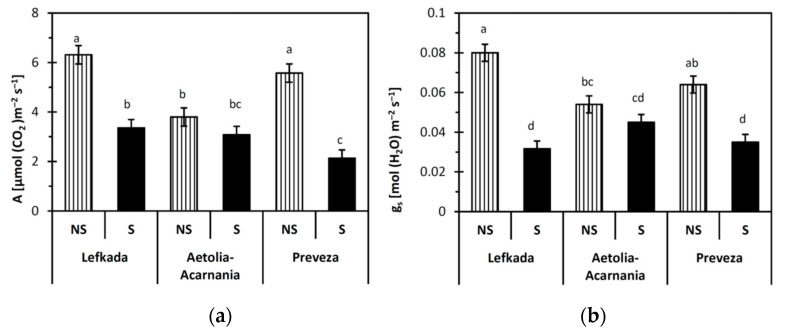
(**a**) Net photosynthetic rate (A), **(b)** stomatal conductance (g_s_) of plants from three *Aegilops triuncialis* populations grown in serpentine (S) and non-serpentine (NS) soils. Values represent means ± SE (*n* = 5). Different letters on columns indicate significant differences for the same parameter (*p*< 0.05).

**Figure 2:**
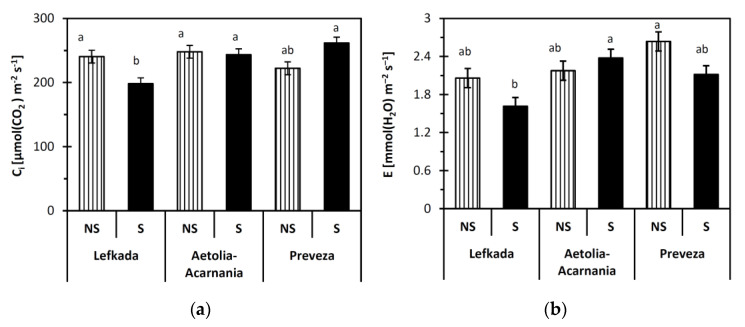
(**a**) Internal CO_2_ concentration (C_i_), (**b**) transpiration rate (E) of plants from three *Aegilops triuncialis* populations grown in serpentine (S) and non-serpentine (NS) soils. Values represent means ± SE (*n* = 5). Different letters on columns indicate significant differences for the same parameter (*p* < 0.05).

**Figure 3:**
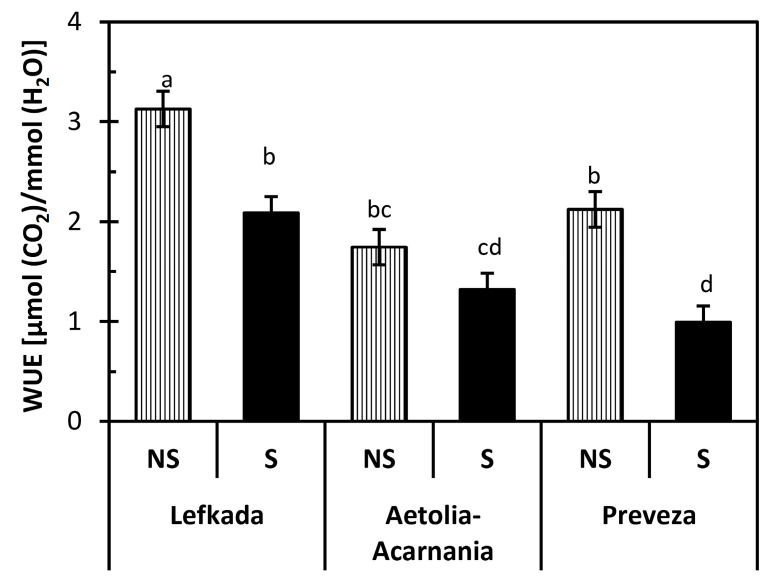
Instantaneous water use efficiency (WUE) of plants from three *Aegilops triuncialis* populations grown in serpentine (S) and non-serpentine (NS) soils. Values represent means ± SE (*n* = 5). Different letters on columns indicate significant differences (*p* < 0.05).

Data analysis revealed that population (Lefkada, Preveza, Aetolia-Acarnania) was a significant (*p* < 0.05) predictor of the net photosynthetic rate (A), transpiration rate (E), and instantaneous water use efficiency (WUE). On the other hand, the type of the soil (S, NS) significantly affected (*p* < 0.05) stomatal conductance g_s,_ A, E, and WUE. In addition, the interaction between population and soil type was significant (*p* < 0.05) for all gas exchange parameters except WUE. The response of the Lefkada and Preveza populations was different in the two types of soil considered in all gas exchange parameters except E ( Figure 1 Figure 2 and Figure 3). The E parameter in the Lefkada population was similar between the S and NS soils. In contrast, the performance of the Aetolia-Acarnania population was similar in the two soil types (*p* > 0.05).

All gas exchange parameters measured for both Lefkada and Preveza populations were higher in NS than S soil except C_i_ ( Figure 1 and Figure 2). Net photosynthetic rate and g_s_ in the S soil for plants from Lefkada decreased by 46% and 60%, respectively, compared to NS soils. Reduction in A and g_s_ for plants from Preveza was 61% and 45%, respectively (Figure 1a,b). The WUE presented the same decrease in Lefkada (33%) and Preveza (33%) in S compared to NS soil (Figure 3).

### 2.3. Chlorophyll Fluorescence Parameters and Proline

Population was a significant (*p* < 0.05) predictor for the initial fluorescence intensity (Fo), the maximal fluorescence intensity (Fm), the ratios Fv/Fm and Fv/Fo, and the performance index (PI). On the other hand, the type of the soil (S, NS) significantly affected (*p* < 0.05) the variable fluorescence (Fv), Fv/Fm, Fv/Fo, and PI. In addition, the interaction between population and type of the soil was significant (*p* < 0.05) for all chlorophyll fluorescence parameters except Fv.

Most of the chlorophyll fluorescence indices for all populations were higher in NS than S soils. More specifically, maximum photochemical efficiency of PSII (Fv/Fm), as well as the water splitting system of the donor side of PSII (Fv/Fo) and finally the performance index highly decreased in S compared to NS soils in all populations (Table 2). The most pronounced decreased of Fv/Fo was recorded in the Preveza population among S and NS soils. Additionally, PI was highly decreased by 81% in S soils compared to NS (Table 2).

Population and soil type (S, NS) were significant (*p* < 0.05) predictors of proline content. In addition, the interaction between population and soil was significant (*p* < 0.05). Proline content was significantly higher in plants growing in S compared to NS soils (*p* < 0.05). The highest difference (up to 73%) between S and NS soils was found in the Preveza population (Table 2).

### 2.4. Plant Tissue Nutrient Concentrations

**Figure 4:**
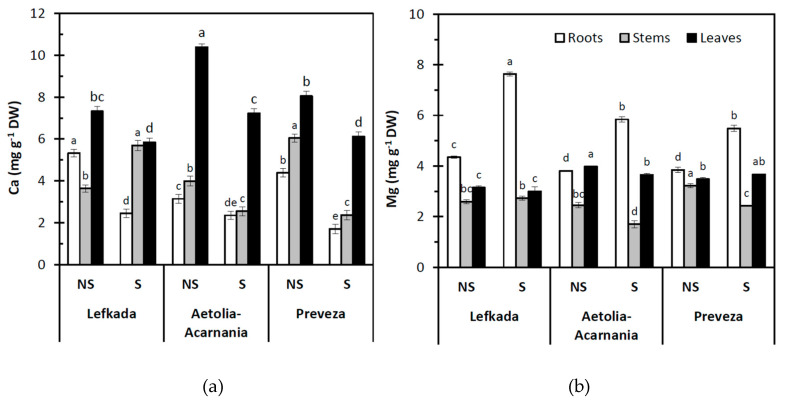
Concentration of (**a**) Ca and (**b**) Mg in the roots, stems, and leaves of plants from three *Aegilops triuncialis* populations grown in serpentine (S) and non-serpentine (NS) soils. Values represent means ± SE (*n* = 5). Different letters for the same plant part indicate significant differences (*p* < 0.05).

**Figure 5:**
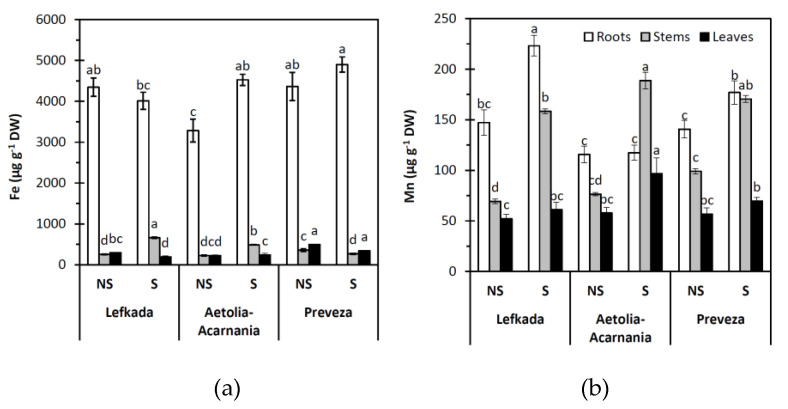
Concentration of (**a**) Fe and (**b**) Mn in the roots, stems, and leaves of plants from three *Aegilops triuncialis* populations grown in serpentine (S) and non-serpentine (NS) soils. Values represent means ± SE (*n* = 5). Different letters for the same plant part indicate significant differences (*p* < 0.05).

Population was a significant (*p* < 0.05) predictor of Ca, Mg, Fe, and Mn concentration in plant tissues (leaves, stems, roots). On the other hand, the type of the soil (S, NS) significantly affected (*p* < 0.05) concentration of Ca, Mg, Fe, and Mn in leaves, stems, and roots except Mg concentration in the leaves. In addition, the interaction between population and soil was significant (*p* < 0.05) for all these parameters (Figure 4 and Figure 5).

The lowest concentrations of Ca in roots, stems, and leaves were found in the S soil in contrast to the NS soil within each plant population, except for Ca in stems from the Lefkada population. Calcium concentrations were higher in roots and leaves of the NS soil in all plant populations compared to S soil. The differences between the two soil types were significant (*p* < 0.05) in all plant parts (Figure 4a). The highest concentrations of Mg in the roots were recorded in the S soil. Difference in Mg concentration in roots and stems between S and NS soils was significant for all three populations (*p* < 0.05). For Mg concentrations in leaves, significant differences (*p* < 0.05) between the two soil types were detected only in the Aetolia-Acarnania population (Figure 4b).

The Fe concentrations were much higher in roots than in stems and leaves. The lowest value of Fe in roots was reported in the Aetolia-Acarnania population growing in the NS soil (*p* < 0.05) (Figure 5a). The higher Mn concentrations were measured in roots (except Aetolia-Acarnania plant population in S soil) and the lowest in leaves in all populations and both soil types. However, in all plant populations, Mn concentrations were higher in S than in the NS soil (Figure 5b).

**Figure 6:**
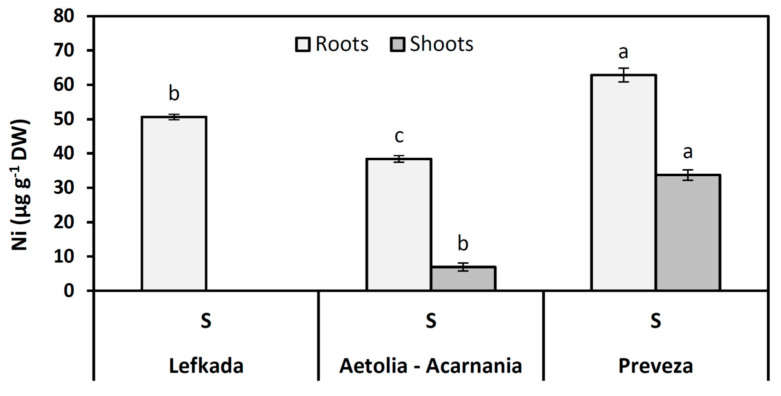
Concentration of Ni in the shoots and roots of plants from three *Aegilops triuncialis* populations grown in serpentine soil (S). Values represent means ± SE (*n* = 5). Different letters for the same plant part indicate significant differences (*p* < 0.05).

Nickel was detected only in serpentine soils, and its concentration was much higher in roots than in shoots. In shoots of plants from the Lefkada population, no Ni concentrations were detected (Figure 6).

Population was a significant (*p* < 0.05) predictor for the Ca/Mg ratio in leaves, stems, and roots. On the other hand, the soil type (S, NS) significantly affected (*p* < 0.05) the Ca/Mg ratio in leaves and roots. In addition, the interaction between population and soil was significant (*p* < 0.05) in all plant parts but leaves (Table 3).

## 3. Discussion

The results of the current study demonstrate a) differential photosynthetic performance under S and NS soils for all three *Ae. triuncialis* populations tested, b) the three populations responded differently to serpentine stress and c) only the population Aetolia-Acarnania seems to tolerate the serpentine stress and high Ni concentration.

The reduced net photosynthetic rate and g_s_ in the serpentine soil for the Lefkada and Preveza populations indicate a possible involvement of the high Ni concentration that apparently restricts A and g_s_. The same variations in A and g_s_ under heavy metal stress have been reported [47]. On the other hand, the similar A and g_s_ in S and NS soil of the Aetolia-Acarnania population indicate no effects of S-stress on the photosynthetic system of these plants. Nickel toxicity (serpentine stress) very often results in declining transpiration rate and water content due to initiation of stomata closure, which is among the primary effects of heavy metals on plant physiology [48,49]. Only one out of the three populations tested expressed decreased transpiration rate under serpentine stress, which rather suggests that that stomatal limitation is not the main factor affecting A and E [50,51]. Likewise, serpentine stress had no effect on the intercellular CO_2_ concentration in other studies [51,52]. Heavy metals are known to induce non-specific inhibition of photosynthesis involving direct and indirect mechanisms [53]. The reduced rate of photosynthesis is related to disrupted chloroplast structure, blocked chlorophyll synthesis, disordered electron transport, inhibited activities of the Calvin cycle enzymes, and CO_2_ deficiency caused by stomatal closure [54]. Under serpentine stress, other factors (e.g., specific environmental conditions, lower activity of photosynthetic enzymes, allocation pattern) or a combination of them may regulate the photosynthetic mechanism in plants from the Aetolia-Acarnania population [46,55]. Papazoglou and coworkers [56] found that the A of the species *Arundo donax* L. was not affected by Ni and cadmium (Cd) concentrations, indicating that its photosystem was not harmed, and showed a strong tolerance to heavy metals. Other authors found that *Picea glehnii* when grown under Ni stress did not decrease its photosynthetic rate because it can maintain low Ni concentrations, and ectomycorrhizal symbiosis may exclude negative Ni effects [57]. Perhaps similar symbiotic associations occur in the root system of barbed goatgrass plants grown in the serpentine soil of our study that impede the uptake of excess Ni. Specific plant species such as *Cenococcum geophilum* Fr. and *Quercus garryana* can survive in serpentine soil by symbiosis with microbes [58,59,60,61].

The water use efficiency and consequently the productivity was different among the three populations. The performance of Lefkada and Preveza populations was higher without stress, while that of Aetolia-Acarnania maintained stable WUE in both soil types. The Aetolia-Acarnania population may have evolved a mechanism to withstand the serpentine stress [62], and/or is preadapted to grow under these specific conditions [22]. This result was also verified from the shoot/root ratio, which was more or less stable in the Aetolia-Acarnania population (NS= 3.84, S = 3.67), while it decreased 2-fold in the other two populations. The toxicity of heavy metals (copper, zinc, lead, nickel, chromium, and cadmium) induces changes in multiple forms of peroxidases leading to shoot and root growth depression [63].

Serpentine soils exhibit distinct chemical and physical properties and are generally regarded as contaminated soils. Heavy metal stress has direct and indirect effects on plant growth and development and very often excess heavy metal-induced mineral nutrient disturbances [44,48,53,64,65,66]. In the present study, serpentine soil had the higher concentration of Ni (53.22ppm) and Mg and lower Ca and Ca/Mg ratio compared to NS. The pH of the serpentine soil is lower than 6.7, and the Ni compounds present in soil are relatively soluble [67]. The nickel stress influenced, as discussed above, the photosynthetic performance and presumably the plant nutrient status [62].

Excess Ni increased proline concentration in all populations, and this increase was more pronounced in Aetolia-Acarnania. Hence, proline, which is common in higher plants and accumulates in large quantities in response to environmental stress, supports plants to maintain osmotic regulation and homeostasis [68]. Accumulation of proline in response to excess Ni has been found in many plant species such as cabbage, soybean, pea, wheat, and rice [44]. Therefore, proline accumulation can be used as a marker to test the level of heavy metal pollution [69,70].

The maximum photochemical efficiency (Fv/Fm) significantly declined in S compared to NS soils, showing an impairment of the primary photochemical efficiency of the photosynthetic apparatus. The decreased photosynthetic efficiency is probably due to high accumulation of Ni in serpentine soils, which reduced the chlorophyll content causing diminution of the pigment content [65,71]. Since Ni affects the PSII photochemistry, it is plausible to argue that the primary photochemistry is directly inhibited by Ni. The reduction of Fv/Fo was mainly due to the decrease of variable fluorescence indicating Ni perturbation on the acceptor side of PSII [72]. Overall, our results demonstrate that the *Ae. triuncialis* plants from the Aetolia-Acarnania population had a lower inhibition of the donor (Fv/Fo) of PSII reaction centers (RCs), maximum photochemical efficiency, and higher performance index compared to Preveza and Lefkada populations.

Our results show that Ni accumulated more in the roots than in the aboveground parts as the translocation factor decreased significantly. A similar decrease in the translocation of Ni to the aboveground parts was observed in previous studies on Poaceae [73] and plant species grown on contaminated soils [51]. It has been found that many plants grown under heavy metal stress accumulated heavy metals in the root system and translocated them to the aboveground parts [74]. Nickel accumulation in the root system, together with/or translocation rate to the above-plant part, is a self-protection mechanism of tolerant plant species under Ni stress to reduce toxicity of heavy metals to the leaves [30,46,51]. Moreover, the tolerant species to serpentine stress can restrict Ni concentration in their leaves [36].

It is well established that Ni can interfere with the uptake of some other trace elements leading to an imbalance in nutrient uptake, transport, and use. In the presence of Ni, the contents of mineral nutrients in plant organs may increase, decrease, or remain unaffected [71]. One of the probable mechanisms for decreasing the uptake of macro- and micro-nutrients by Ni relies on the competition for common binding sites due to comparable ionic radii of Ni and other cations. Such mechanisms may have operated when the uptake of Mg, Fe, and Zn decreased in the presence of Ni [42,75]. Our data demonstrated that increased Ni concentration in roots did not influence Mg and Fe uptake by roots; however, it influenced their translocation to upper parts via competition and then induced deficiencies of these elements in aboveground plant parts [36]. The decline of nutrient uptake may also result from Ni-induced metabolic disorders that affect the structure and enzyme activities of cell membranes [54]. Elevated levels of Ni in soil may cause various physiological alterations and diverse toxicity symptoms such as chlorosis and necrosis in a variety of plant species [76]. The reduced uptake of Mg and Fe is one of the prime causes of chlorosis induced by excess Ni [77]. However, the nickel stress differentially influences the translocation rate of the three populations to stems and leaves. Our populations did not show any obvious symptoms of toxicity when growing in the S soil. The control (NS) plants were distinguished by S plants from the higher growth which was more apparent in the Lefkada and Preveza populations. The increased Ni uptake in roots of the Lefkada and Aetolia-Acarnania populations in the serpentine soil is accompanied with an increased accumulation of Fe and Mg and Mg in shoots and decreased accumulation of Fe, Mg ions in leaves compared to non-serpentine soils. The excess Ni in serpentine soils caused Ca deficiency in all populations. Nickel causes a severe decrease in Ca in plant tissues, and since Ca is necessary for the development of the cell wall and the maintenance of the membrane structure, Ni may also indirectly affect plant growth [72]. The Ca/Mg ratio for the above-plant part for Aetolia-Acarnania and Lefkada populations was >1.0 and close to 1 (0.98) for the Preveza population. Plant species growing in serpentine soils express limited uptake of Mg and an increased capacity to absorb Ca; thus, the lower available Ca is more efficiently utilized [30,46]

Excess Ni in serpentine soils increased Mn uptake and accumulation in roots of all three populations. Mn translocation to aboveground parts remained stable except for the Aetolia-Acarnania population. The increased translocation in plants from the Aetolia-Acarnania population suggests that photosynthesis is not inhibited in the presentence of increased Ni (see Figure 1 and Figure 6). The Mn deficiency in plants decreases growth and biomass due to lower net photosynthetic efficiency, and a decrease in chlorophyll content and chlorophyll biosynthesis [78,79,80,81]. Indeed, both Lefkada and Preveza populations presented lower A and WUE.

The iron concentrations in aboveground tissues in serpentine soils was increased compared to non-serpentine soil except Preveza population and that is probably the reason that Preveza in serpentine soil had a sharp decrease in maximum photochemical efficiency. It is well known that Fe, Ca, and Mg cations have major roles in regulating (directly or indirectly) photosynthetic efficiency. In contrast to previous studies that suggest no effect or antagonistic effects of the Ni status on Fe uptake [82], we found a synergistic effect on Fe uptake and accumulation in the roots of plants growing in serpentine soils. Iron is an essential trace element required for respiration, photosynthesis, and many fundamental biological redox reactions. In the photosynthesis apparatus, Fe protects PSII from photoinhibition that occurs under Fe deficiency, and its supplement maintains the photosynthetic electron transport. Therefore, the observed decrease of Fe and Mg accumulation in the leaves especially in plants from the Preveza population could explain the decreased photochemical efficiency of PSII in this population. Very often the high uptake of Ni induced a decline in water content of many plant species [83].

In the current study we demonstrated that the three barbed goatgrass populations presented low or no accumulation of Ni in leaves and stems, suggesting restricted nickel transportation to shoots, and hence they can be considered as metal excluders. The population from Aetolia-Acarnania expressed a remarkable response to heavy metal stress compared to two other populations and hence can be further explored to gain a deeper insight of the involved physiological and molecular mechanisms. The variability of the response to serpentine soil stress among the three barbed goatgrass populations tested highlights the need for including additional populations of the above species from other geographic areas in future studies. Adding additional important *Aegilops* species in these studies would be further broaden the scope and generate much needed data on the stress physiology of crop wild relatives.

## 4. Materials and Methods

### 4.1. Study Area and Climate

The study was conducted in spring 2017 in the farm of Aristotle University of Thessaloniki, Northern Greece (long. 40°31’91’’, lat. 23°59’58’’, altitude 6 m a.s.l.). The climate of the study area is characterized as Cfa in the Köppen–Geiger system (http://www.en.climate-data.org) and as Mediterranean semi-arid with cold winters, hot summers, and a long dry period according to the bioclimatogram of Emberger [84]. The mean annual temperature of the site was estimated at 15.5 °C and the mean annual rainfall at 443 mm for the period 1987–2017.

Microclimatic conditions in the study areas were measured under ambient conditions when gas exchange measurements were taken. Air temperature (Ta) and relative humidity (RH) were acquired using a Novasima MS1 microclimatic sensor (Novatron Scientific Ltd. Horsham. UK), while vapor pressure deficit (VPD) was calculated according to Abtew and Melesse [85]. Ta, RH, and VPD were 24.3 ± 0.2 °C, 31.5 ± 1.9%, and 2.01 ± 0.24 kPa, respectively. The values of VPD, RH, and Ta given are averages of six measurements.

### 4.2. Plant Materials

Three populations of *Aegilops triuncialis* (barbed goatgrass) originating from Western Greece, non-serpentine areas [86], obtained from the Greek “GeneBank”, were used (Table 4).

### 4.3. Chemical Analysis of Soil Samples

The soil samples were air-dried, passed through a 2 mm sieve, and analyzed for specific chemical properties. Soil pH was measured in the saturated paste [87]. Organic matter (OM) content was determined by the Walkley–Black method [88]. Total soil N was measured using the Kjeldahl method [89], while available P was determined by the Olsen method [90]. Exchangeable potassium, calcium, magnesium, and sodium (Na) were extracted using 1N CH_3_COONH_4_ pH = 7 [91] and determined by atomic absorption spectroscopy. The available forms of iron, copper, zinc, manganese, nickel, chromium, cadmium, cobalt (Co), and lead (Pb) were extracted using DTPA (0.005 M DTPA. 0.1 M TEA and 0.01 M CaCl_2_, pH = 7.3) [92] and determined by atomic absorption spectroscopy.

### 4.4. Growth Conditions

The following two soil types were considered: a) surface soil (0–20 cm) from the farm of the Aristotle University of Thessaloniki and b) serpentine soil that was collected from the slopes of Mount Chortiatis (40°34′39, 23°05′52, 750 m a.s.l.) about 15 km away from the University farm. Three seeds of each population were sown in each of 36 pots (10 × 10 × 20 cm) filled with either soil type (a) or (b). Hence, 15 pots were randomly assigned to soil type (a) and 18 to soil type (b). Details for the two soil types are given in Table 1. The most vigorous seedling out of the three was kept in every pot. All plants were irrigated frequently with the Hoagland solution so that the soil in the pots was always near the field capacity. Every second week all pots were randomized within each treatment. At the end of the experimental period the aboveground (herbage) and underground parts of plants from all treatments were harvested.

### 4.5. Gas Exchange Measurements

For each population of *Ae. triuncialis* and soil type treatment, we estimated the following leaf gas-exchange parameters: (a) net photosynthetic rate, (b) stomatal conductance, (c) transpiration rate, and (d) intercellular CO_2_ concentration. All measures were taken under ambient conditions (380 μmol (CO_2_) mol^−1^, 1,500 μmol (photon) m^−2^ s^−1^) employing a portable photosynthesis system (LCpro-SD, ADC Bioscientific Ltd., Hoddesdon, UK) from 11:00 to 13:00 h on five mature, intact, fully expanded upper leaves. The instantaneous water use efficiency was calculated from the ratio A/E [55].

### 4.6. In Vivo Chlorophyll Fluorescence Measurements

In vivo PSII chlorophyll fluorescence was measured on the upper leaf surface on five mature, fully expanded leaves by a modulated (1.6 kHz), low intensity beam from light emitting diodes (excitation wavelength 655nm, detection above 700nm) using a portable pulse-amplitude-modulated fluorometer (PEA-Hansatech; Walz, Germany). The following fluorescence parameters were measured: the initial fluorescence intensity when all reactions centers are open, the maximal fluorescence intensity when all reactions are closed, the variable fluorescence, the ratios Fv/Fm and Fv/Fo, and performance index [93].

### 4.7. Determination of Proline Content

Sampled fully developed young leaves of five *Ae. triuncialis* plants from the six treatments were cut into small pieces as described by Giannakoula and Ilias [94]. From these samples, approximately 0.3 g was weighed and placed separately into glass vials containing 10 mL of 80% (*v/v*) ethanol. Then, they were heated at 60 °C for 30 min, and the extracts were filtered and diluted with 80% (*v/v*) ethanol up to 20 mL. In these extracts, the free proline concentration was determined by the acid-ninhydrin reagent method. Approximately 1 g of ninhydrin was added to 500 mL of dense H_2_SO_4_. Then, 2 mL of the acid-ninhydrin and 2 mL of the aqueous alcohol extract were transferred into test tubes. The test tubes were covered by glass marbles to minimize evaporation and maintained at 95 °C for 60 min in a water bath. After this time, they were allowed to cool at room temperature. Finally, 4 mL of toluene was added to each replicate of the sample and mixed thoroughly. After separation of solution layers, the toluene layer was carefully decanted, placed in glass cuvettes, and its absorption was determined at 518 nm.

### 4.8. Plant Tissue Nutrient Analysis

Selected plants were separated into leaves, stems, and roots. Plant tissues were washed once with tap and twice with distilled water, dried at 72 °C until constant weight, and finally ground to a fine powder. Then, 0.5 g subsamples were ashed at 500 °C for 4 h [95]. The ash was dissolved in 6 N hydrochloric acid (HCl), filtered, and analyzed for total calcium, magnesium, iron, manganese, and nickel using atomic absorption spectroscopy.

### 4.9. Statistical Analysis

Generalized linear models were used to assess the effects of serpentine and non-serpentine soils on gas exchange parameters (A, g_s_, C_i_, E, WUE), proline content, chlorophyll fluorescence parameters, and chemical analysis (nutrition) of three wild population of *Ae. triuncialis*. Estimated marginal means for the factors were calculated with pairwise contrasts and an adjustment for multiple comparisons of Bonferroni (a = 0.05). All statistical analyses were carried out with the SPSS® statistical software v. 25.0 (SPSS Inc., Chicago, IL, USA).

## 5. Conclusions

The toxicity of Ni in plants has become a worldwide problem threatening sustainable agriculture. Following a common garden experimental approach, we demonstrated that three Greek barbed goatgrass populations presented different performance under serpentine and non-serpentine soils, and hence our hypothesis is rejected. The serpentine stressful conditions negatively influenced only the plants from populations of Lefkada and Preveza but not those of Aetolia-Acarnania. The barbed goatgrass population from Aetolia-Acarnania presented a remarkable response to serpentine soil/heavy metal stress maintaining photosynthetic performance and nutrition uptake and hence can be characterized as a serpentine tolerant population. The adaptive and plastic mechanisms involved in this response should be further explored to identify possible genes involved in alleviating the Ni toxicity. A combination of plant breeding and genomic approaches can be considered. In addition, the Aetolia-Acarnania population can be exploited as a possible source of genes for increasing nutrition uptake and reducing toxic metal accumulation in crops. A first approach should consider wheat breeding programs for improving the abiotic stress tolerance and helping to produce wheat varieties more adapted to heavy metal and especially Ni stress. Screening of the response of additional important *Aegilops* species may also be considered to broaden the list of crop wild relatives that can be used in breeding programs regarding major crops. The first step should involve systematic and comprehensive in situ and ex situ conservation approaches to ensure the availability of CWR genetic resources. Last but not least, more fieldwork is required to understand function and sustainability of plant communities involving goatgrass species in habitats with challenging soil and climatic conditions.

## Figures and Tables

**Table 1:** Soil chemical properties of non-serpentine (NS) and serpentine (S) soils used in the experimental pots.

	S	NS
pH	6.59	7.57
C (%)	1.82	1.26
Organic matter (%)	3.16	2.16
N (%)	0.28	0.15
P (ppm) ^a^	11.91	9.41
Ca (ppm)	1079	2302
Mg (ppm)	454.85	240.14
K (ppm)	158.2	421.2
Na (ppm)	26.6	52.9
Ca/Mg	2.37	9.59
Cu (ppm)	1.78	4.97
Fe (ppm)	30.21	5.24
Zn (ppm)	1.07	1.92
Mn (ppm)	34.63	9.23
Cr (ppm)	0.03	0.005
Ni (ppm)	53.22	1.51
Cd (ppm)	0.019	-
Co (ppm)	0.55	-
Pb (ppm)	0.88	-

^a^ ppm: parts per millio*n* = mg kg^−1^.

**Table 2:** Chlorophyll fluorescence parameters (Fo, Fm, Fv, and the ratios Fv/Fm and Fv/Fo), performance index (PI), and proline content (mmol g^−1^ FW) from three *Aegilops triuncialis* populations grown in serpentine (S) and non-serpentine (NS) soils. Values represent means ± SE (*n* = 5). Different letters in each column indicate significant differences (*p* < 0.05).

Population	Soil Type	Average ± SE						
		Fo	Fm	Fv	Fv/Fo	Fv/Fm	PI	Proline Content
Lefkada	NS	443 ± 14b	1833 ± 75a	1390 ± 38a	3.13 ± 0.36a	0.75 ± 0.02a	1.23 ± 0.50b	36 ± 0.6b
	S	479 ± 22b	1611 ± 54b	1132 ± 41b	2.37 ± 0.21b	0.70 ± 0.005b	0.5 ± 0.07d	42 ± 0.9a
Aetolia-Acarnania	NS	417 ± 9c	1723 ± 68b	1326 ± 41a	3.17 ± 0.31a	0.74 ± 0.004a	1.24 ± 0.05b	27 ± 0.8c
	S	491 ± 17b	1766 ± 62b	1275 ± 40ab	2.59 ± 0.09b	0.72 ± 0.003b	0.63 ± 0.03c	31 ± 1.0b
Preveza	NS	556 ± 11a	2093 ± 71a	1537 ± 39a	2.76 ± 0.15a	0.72 ± 0.005b	2.06 ± 0.21a	26 ± 0.7c
	S	445 ± 19b	1309 ± 65c	864 ± 29c	1.94 ± 0.11c	0.65 ± 0.003c	0.38 ± 0.08c	45 ± 1.2a

**Table 3:** Ratio of Ca/Mg in roots, stems, and leaves of plants from three *Aegilops triuncialis* populations growth in serpentine (S) and non-serpentine (NS) soils (*n* = 5). Different letters in each column indicate significant differences (*p* < 0.05).

Population	Soil Type	Ca/Mg ± SE		
		Root	Stem	Leaf
Lefkada	NS	1.22 ± 0.03a	1.40 ± 0.06b	2.33 ± 0.117a
	S	0.32 ± 0.03c	2.12 ± 0.22a	1.95 ± 0.10b
Aetolia-Acarnania	NS	0.83 ±0.06b	1.62 ± 0.05b	2.62 ± 0.08a
	S	0.40 ± 0.03c	1.50 ± 0.014b	1.99 ± 0.13b
Preveza	NS	1.14 ± 0.03a	1.88 ± 0.07a	2.31 ± 0.07a
	S	0.31 ± 0.04c	0.98 ± 0.09c	.67 ± 0.073b

**Table 4:** List of *Aegilops triuncialis* populations used in the study.

Collection Sites	Altitude (m)	Longitude (N)	Latitude (E)	Code ^2^
Lefkada, Karya	430	38^o^45’29’’	20^o^38’56’’	GRC805/04
Aetolia–Akarnania, Τhermo	480	38^o^34’15’’	20^o^40’1’’	GRC987/04
Preveza, Skiados Polystafyllo ^1^	543	39^ο^20’33’’	20^ο^47’17’’	GRC1255/04 ^1^

^1^ The first name is the prefecture and the second the village. ^2^ Collection code of Greek “GeneBank”.

## Data Availability

The data presented in this study are available in figures and tables provided in the manuscript.

## References

[1] Maxted N., White K., Valkoun J., Konopka J., Hargreaves S. (2008). Towards a conservation strategy for Aegilops species. Plant Genet. Resour..

[2] Zair W., Maxted N., Amri A. (2018). Setting conservation priorities for crop wild relatives in the Fertile Crescent. Genet. Resour. Crop Evol..

[3] Godfray H.C.J., Beddington J.R., Crute I.R., Haddad L., Lawrence D., Muir J.F., Pretty J., Robinson S., Thomas S.M., Toulmin C. (2010). Food security: The challenge of feeding 9 billion people. Science.

[4] Guzzon F., Müller J.V., Abeli T., Cauzzi P., Ardenghi N.M.G., Balestrazzi A., Rossi G., Orsenigo S. (2015). Germination requirements of nine European Aegilops species in relation to constant and alternating temperatures. Acta Bot. Gall..

[5] Benlioğlu B., Adak M.S. (2019). Importance of crop wild relatives and landraces genetic resources in plant breeding programmes. J. Exp. Agric. Int..

[6] Derneg D. (2010). Ecosystem profile: Mediterranean basin biodiversity hotspot. CEPF Crit Ecosyst. Partnersh. Fund.

[7] Perrino E.V., Musarella C.M., Magazzini P. (2020). Management of grazing Italian river buffalo to preserve habitats defined by Directive 92/43/EEC in a protected wetland area on the Mediterranean coast: Palude Frattarolo, Apulia, Italy. Euro-Mediterr. J. Environ. Integr.

[8] Ortiz R. (2015). The importance of crop wild relatives, diversity, and genetic potential for adaptation to abiotic stress-prone environments. Crop Wild Relatives and Climate Change.

[9] Von Wettberg E., Davis T.M., Smýkal P. (2020). Editorial: Wild Plants as Source of New Crops. Front. Plant Sci..

[10] Jarvis A., Lane A., Hijmans R.J. (2008). The effect of climate change on crop wild relatives. Agric. Ecosyst. Environ..

[11] FAO A. (2007). Adaptation to climate change in agriculture, forestry and fisheries: Perspective, framework and priorities.

[12] Raizada P., Singh A., Raghubanshi A.S. (2009). Comparative response of seedlings of selected native dry tropical and alien invasive species to CO_2_ enrichment. J. Plant Ecol..

[13] Ostrowski M.F., Prosperi J.M., David J. (2016). Potential implications of climate change on aegilops species distribution: Sympatry of these crop wild relatives with the major european crop triticum aestivum and conservation issues. PLoS ONE.

[14] Zair W., Maxted N., Brehm J.M., Amri A. (2021). Ex situ and in situ conservation gap analysis of crop wild relative diversity in the Fertile Crescent of the Middle East. Genet. Resour. Crop Evol..

[15] Maxted N., Kell S. (2009). Establishment of a Global Network for the In Situ Conservation of Crop Wild Relatives: Status and Needs.

[16] Vincent H., Wiersema J., Dobbie S., Kell S.P., Fielder H., Castañeda Alvarez N.P., Guarino L., Eastwood R., Leόn B., Maxted N. (2012). A prioritised crop wild relative inventory as a first step to help underpin global food security. Biol. Conserv..

[17] Perrino E., Perrino P. (2020). Crop wild relatives: Know how past and present to improve future research, conservation and utilization strategies, especially in Italy: A review. Genet. Resour. Crop Evol..

[18] Perrino E.V., Wagensommer R.P. (2021). Crop Wild Relatives (CWR) priority in Italy: Distribution, ecology, in situ and ex situ conservation and expected actions. Sustainability.

[19] Garcia R.M., Parra-Quijano M., Iriondo J.M. (2017). Identification of ecogeographical gaps in the Spanish Aegilops collections with potential tolerance to drought and salinity. PeerJ.

[20] Loureiro I., Escorial M.C., Garcia-Baudin J.M., Chueca M.C. (2007). Hybridization between wheat (Triticum aestivum) and the wild species Aegilops geniculata and A. biuncialis under experimental field conditions. Agric. Ecosyst. Environ..

[21] Van Slageren M.W. (1994). Wild Wheats: A Monograph of Aegilops L. and Amblyopyrum (Jaub. & Spach) Eig (Poaceae).

[22] Meimberg H., Milan N.F., Karatassiou M., Espeland E.K., McKAY J.K., Rice K.J. (2010). Patterns of introduction and adaptation during the invasion of Aegilops triuncialis (Poaceae) into Californian serpentine soils. Mol. Ecol..

[23] Harrison S. (1999). Local and regional diversity in a patchy landscape: Native, alien, and endemic herbs on serpentine. Ecology.

[24] Harrison S., Rice K., Maron J. (2001). Habitat patchiness promotes invasion by alien grasses on serpentine soil. Biol. Conserv..

[25] Kruckeberg A.R. (1985). California Serpentines: Flora, Vegetation, Geology, Soils, and Management Problems.

[26] Perrino E.V., Wagensommer R.P., Medagli P. (2014). Aegilops(Poaceae) in Italy: Taxonomy, geographical distribution, ecology, vulnerability and conservation. Syst. Biodivers..

[27] Kim J.M., Shim J.K. (2008). Toxic effects of serpentine soils on plant growth. J. Ecol. Environ..

[28] O’Dell R.E., James J.J., Richards J.H. (2006). Congeneric serpentine and nonserpentine shrubs differ more in leaf Ca: Mg than in tolerance of low N, low P, or heavy metals. Plant Soil.

[29] Asemaneh T., Ghaderian S.M., Baker A.J.M. (2007). Responses to Mg/Ca balance in an Iranian serpentine endemic plant, Cleome heratensis (Capparaceae) and a related non-serpentine species, C. foliolosa. Plant Soil.

[30] Kazakou E., Adamidis G.C., Baker A.l.M., Reeves R.D., Godino M., Dimitrakopoulos P.G. (2010). Species adaptation in serpentine soils in Lesbos Island (Greece): Metal hyperaccumulation and tolerance. Plant Soil.

[31] Muchuweti M., Birkett J.W., Chinyanga E., Zvauya R., Scrimshaw M.D., Lester J.N. (2006). Heavy metal content of vegetables irrigated with mixtures of wastewater and sewage sludge in Zimbabwe: Implications for human health. Agric. Ecosyst. Environ..

[32] Abedi T., Mojiri A. (2020). Cadmium uptake by wheat (*Triticum aestivum* L.): An over view. Plants.

[33] Grime J.P. (1988). The CSR model of primary plant strategies—Origins, implications and tests. Plant Evolutionary Biology.

[34] Von Wettberg E.J., Ray-Mukherjee J., D’Adesky N., Nesbeth D., Sistla S., Rajakaruna N., Boyd R.S., Harris T. (2014). The evolutionary ecology and genetics of stress resistance syndrome (SRS) traits: Revisiting Chapin, Autumn and Pugnaire (1993). Plant Ecology and Evolution in Harsh Environments.

[35] Bini C., Maleci L. (2014). The “serpentine syndrome”(H. Jenny, 1980): A proxy for soil remediation. Eqa-Int. J. Environ. Qual..

[36] Saez Ondarra A.M. Local Adaptation to Serpentine Soils: A Review of Adaptive Traits and Underlying Genetic Bases. 2020. http://hdl.handle.net/10810/49089.

[37] Kumar A., Memo M., Mastinu A. (2020). Plant behaviour: An evolutionary response to the environment?. Plant Biol..

[38] Jenny H. (1980). Ecosystems and soils. The Soil Resource: Origin and Behavior.

[39] Brady K.U., Kruckeberg A.R., Bradshaw H.D. (2005). Evolutionary ecology of plant adaptation to serpentine soils. Annu. Rev. Ecol. Evol. Syst..

[40] Palm E.R., Van Volkenburgh E. (2014). Physiological adaptations of plants to serpentine soil. Plant Ecology and Evolution in Harsh Environments.

[41] Kawai K., Saito H., Kajino H., Nakai W., Nakamura R., Sato K., Okada N. (2019). Leaf water relations and structural traits of four temperate woody species occurring in serpentine and non-serpentine soil. Ecol. Res..

[42] Rahman H., Sabreen S., Alam S., Kawai S. (2005). Effects of nickel on growth and composition of metal micronutrients in barley plants grown in nutrient solution. J. Plant. Nutr..

[43] Seregin I.V., Kozhevnikova A.D. (2006). Physiological role of nickel and its toxic effects on higher plants. Russ. J. Plant Physiol..

[44] Yusuf M., Fariduddin Q., Hayat S., Ahmad A. (2011). Nickel: An overview of uptake, essentiality and toxicity in plants. Bull. Environ. Contam Toxicol.

[45] Adamidis G.C., Aloupi M., Kazakou E., Dimitrakopoulos P.G. (2014). Intra-specific variation in Ni tolerance, accumulation and translocation patterns in the Ni-hyperaccumulator Alyssum lesbiacum. Chemosphere.

[46] Chatzistathis T., Papadakis I.E., Papaioannou A., Dichala O., Giannakoula A., Kostas S., Tziachris P. (2019). Genotypic tolerance of two *Punica granatum* L. cultivars (‘Wonderful’and ‘Acco’) to serpentine stress. Sci. Hortic..

[47] Xu J., Peng S., Wei Z., Jiao X. (2010). Intercellular CO2 concentration and stomatal or non-stomatal limitation of rice under water saving irrigation. Trans. Chin. Soc. Agric. Eng..

[48] Molas J. (1998). Changes in morphological and anatomical structure of cabbage (*Brassica oleracea* L.) outer leaves and in ultrastructure of their chloroplasts caused by an in vitro excess of nickel. Photosynthetica.

[49] Bhalerao S.A., Sharma A.S., Poojari A.C. (2015). Toxicity of nickel in plants. Int. J. Pure Appl. Biosci..

[50] Fu L., Chen C., Wang B., Zhou X., Li S., Guo P., Shen Z., Wang G., Chen Y. (2015). Differences in copper absorption and accumulation between copper-exclusion and copper-enrichment plants: A comparison of structure and physiological responses. PLoS One.

[51] Yang Y., Zhang L., Huang X., Zhou Y., Quan Q., Li Y., Zhu X. (2020). Response of photosynthesis to different concentrations of heavy metals in Davidia involucrata. PLoS One.

[52] Leal-Alvarado D.A., Espadas-Gil F., Saenz-Carbonell L., Talavera-May C., Santamaria J.M. (2016). Lead accumulation reduces photosynthesis in the lead hyper-accumulator Salvinia minima Baker by affecting the cell membrane and inducing stomatal closure. Aquat. Toxicol..

[53] Maksymiec W. (2007). Signaling responses in plants to heavy metal stress. Acta Physiol. Plant..

[54] Seregin I., Ivanov V. (2001). Physiological aspects of cadmium and lead toxic effects on higher plants. Russ. J. Plant Physiol..

[55] Kostopoulou P., Karatassiou M. (2017). *Lotus corniculatus* L. response to carbon dioxide concentration and radiation level variations. Photosynthetica.

[56] Papazoglou E.G., Karantounias G.A., Vemmos S.N., Bouranis D.L. (2005). Photosynthesis and growth responses of giant reed (*Arundo donax* L.) to the heavy metals Cd and Ni. Environ. Int..

[57] Kayama M., Choi D., Tobita H., Utsugi H., Kitao M., Maruyama Y., Nomura M., Koike T. (2006). Comparison of growth characteristics and tolerance to serpentine soil of three ectomycorrhizal spruce seedlings in northern Japan. Trees.

[58] Panaccione D.G., Sheets N.L., Miller S.P., Cumming J.R. (2001). Diversity of cenococcum geophilum isolates from serpentine and non-serpentine soils. Mycologia.

[59] Gonçalves S.C., Martins-Loução M.A., Freitas H. (2009). Evidence of adaptive tolerance to nickel in isolates of Cenococcum geophilum from serpentine soils. Mycorrhiza.

[60] Obase K., Douhan G.W., Matsuda Y., Smith M.E. (2018). Isolation source matters: Sclerotia and ectomycorrhizal roots provide different views of genetic diversity in Cenococcum geophilum. Mycologia.

[61] Moser A.M., Petersen C.A., D’allura J.A., Southworth D. (2005). Comparison of ectomycorrhizas of Quercus garryana (Fagaceae) on serpentine and non-serpentine soils in southwestern Oregon. Am. J. Bot..

[62] Amjad M., Raza H., Murtaza B., Abbas G., Imran M., Shahid M., Naeem M.A., Zakir A., Iqbal M.M. (2020). Nickel toxicity induced changes in nutrient dynamics and antioxidant profiling in two maize (*Zea mays* L.) Hybrids. Plants.

[63] Dimitrijevic M., Petrovic S. (2014). Species variation of Aegilops genus and heavy metal content in plant habitat soil at southern Adriatic localities. Genetika.

[64] Gajewska E., Sklodowska M., Slaba M., Mazur J. (2006). Effect of nickel on antioxidative enzyme activities, proline and chlorophyll contents in wheat shoots. Biol. Plant..

[65] Chen C., Huang D., Liu J. (2009). Functions and Toxicity of Nickel in Plants: Recent Advances and Future Prospects. Clean Soilairwater.

[66] Liu X., Zhang H., Wang J., Wu X., Ma S., Xu Z., Zhou T., Xu N., Tang X., An B. (2019). Increased CO2 concentrations increasing water use efficiency and improvement PSII function of mulberry seedling leaves under drought stress. J. Plant Interact..

[67] Sreekanth T.V.M., Nagajyothi P.C., Lee K.D., Prasad T.N.V.K.V. (2013). Occurrence, physiological responses and toxicity of nickel in plants. Int. J. Environ. Sci. Technol..

[68] Chun S.C., Paramasivan M., Chandrasekaran M. (2018). Proline accumulation influenced by osmotic stress in arbuscular mycorrhizal symbiotic plants. Front. Microbiol..

[69] Theriappan P., Gupta A.K., Dhasarrathan P. (2011). Accumulation of proline under salinity and heavy metal stress in cauliflower seedlings. J. Appl. Sci. Environ. Manag..

[70] Rizvi A., Zaidi A., Ameen F., Ahmed B., AlKahtani M.D., Khan M.S. (2020). Heavy metal induced stress on wheat: Phytotoxicity and microbiological management. RSC Adv..

[71] Sachan P., Lal N. (2017). An overview of nickel (Ni^2+^) essentiality, toxicity and tolerance strategies in plants. Asian J. Biol..

[72] Ouzounidou G., Moustakas M., Symeonidis L., Karataglis S. (2006). Response of wheat seedlings to Ni stress: Effects of supplemental calcium. Arch. Environ. Contam. Toxicol..

[73] Antonkiewicz J., Jasiewicz C., Koncewicz-Baran M., Sendor R. (2016). Nickel bioaccumulation by the chosen plant species. Acta Physiol. Plant..

[74] Chand S., Yaseen M., Rajkumari, Patra D.D. (2015). Application of Heavy metal rich tannery sludge on sustainable growth, yield and metal accumulation by clarysage (*Salvia sclarea* L.). Int. J. Phytoremediation.

[75] Emsley J. (1998). The Elements.

[76] Pandey N., Sharma C.P. (2002). Effect of heavy metals Co^2+^, Ni^2+^ and Cd^2+^ on growth and metabolism of cabbage. Plant Sci..

[77] Piccini D.F., Malavolta E. (1992). Effect of nickel on two common bean cultivars. J. Plant Nutr..

[78] Henriques F. (2004). Reduction in chloroplast number accounts for the decrease in the photosynthetic capacity of Mn-deficient pecan leaves. Plant Sci..

[79] Hebbern C.A., Laursen K.H., Ladegaard A.H., Schmidt S.B., Pedas P., Bruhn D., Schjoerring J.K., Wulfsohn D., Husted S. (2009). Latent manganese deficiency increases transpiration in barley (Hordeum vulgare). Physiol Plant.

[80] Alejandro S., Holler S., Meier B., Peiter E. (2020). Manganese in plants: From acquisition to subcellular allocation. Front Plant Sci.

[81] Alejandro S., Cailliatte R., Alcon C., Dirick L., Domergue F., Correia D., Castaings L., Briat J.F., Mari S., Curie C. (2017). Intracellular distribution of manganese by the trans-golgi network transporter NRAMP2 is critical for photosynthesis and cellular redox homeostasis. Plant Cell.

[82] Dichala O., Therios I., Koukourikou-Petridou M., Papadopoulos A. (2018). Nickel effect on pomegranate cracking, nutrient concentrations, and biochemical parameters of pomegranate peel. HortScience.

[83] Dimkpa C., Svatoš A., Merten D., Büchel G., Kothe E. (2008). Hydroxamate siderophores produced by Streptomyces acidiscabies E13 bind nickel and promote growth in cowpea (*Vigna unguiculata* L.) under nickel stress. Can. J. Microbiol..

[84] Kapsali E.-I., Karatassiou M. (2015). Seasonal changes in leaf tissue rehydration of one annual and two perennial grass forage species induced by bioclimate. Not. Bot. Horti Agrobot. Cluj-Napoca.

[85] Abtew W., Melesse A. (2013). Vapor pressure calculation methods. Evaporation and Evapotranspiration.

[86] Constantinidis T. (2004). The floristic diversity of serpentine in Greece 1. An inventory of the Aliki area (Sterea Ellas, Central Greece). Phyton (Horn).

[87] McLean E.O. (1983). Soil pH and lime requirement. Methods Soil Anal. Part 2 Chem. Microbiol. Prop..

[88] Nelson D.W., Sommers L.E. (1996). Total carbon, organic carbon, and organic matter. Methods Soil Anal. Part 3 Chem. Methods.

[89] Bremner J.M., C M., Page A., Miller R., Keeney D. (1996). Nitrogen-total. Methods of Soil Analysis: Part 3 Chemical Methods.

[90] Olsen S.R., Sommers L.E. (1982). Phosphorus. In page, RH Miller and DR Keeney. Methods of soil analysis. Part 2: Chemical and microbiological properties. Soil Sci. Soc. Am. Inc..

[91] Thomas G.W., Page A.L. (1982). Exchangeable cations. Methods of Soil Analysis: Part 2 Chemical and Microbiological Properties, 9.2.2.

[92] Lindsay W.L., Norvell W.A. (1978). Development of a DTPA Soil Test for Zinc, Iron, Manganese, and Copper. Soil Sci. Soc. Am. J..

[93] Giannakoula A., Therios I., Chatzissavvidis C. (2021). Effect of lead and copper on photosynthetic apparatus in citrus (*Citrus aurantium* L.) plants. The role of antioxidants in oxidative damage as a response to heavy metal stress. Plants.

[94] Giannakoula A.E., Ilias I.F. (2013). The effect of water stress and salinity on growth and physiology of tomato (*Lycopersicon esculentum* Mil.). Arch. Biol. Sci..

[95] Miller R.O., Karla Y.P. (1998). High-temperature oxidation: Dry ashing. Handbook of Reference Methods for Plant Analyses.

